# A Boolean Consistent Fuzzy Inference System for Diagnosing Diseases and Its Application for Determining Peritonitis Likelihood

**DOI:** 10.1155/2015/147947

**Published:** 2015-11-17

**Authors:** Ivana Dragović, Nina Turajlić, Dejan Pilčević, Bratislav Petrović, Dragan Radojević

**Affiliations:** ^1^Faculty of Organizational Sciences, University of Belgrade, Jove Ilića 154, 11000 Belgrade, Serbia; ^2^Medical Faculty of the Military Medical Academy, University of Defense, Crnotravska 17, 11000 Belgrade, Serbia; ^3^Mihajlo Pupin Institute, Volgina 15, 11000 Belgrade, Serbia

## Abstract

Fuzzy inference systems (FIS) enable automated assessment and reasoning in a logically consistent manner akin to the way in which humans reason. However, since no conventional fuzzy set theory is in the Boolean frame, it is proposed that Boolean consistent fuzzy logic should be used in the evaluation of rules. The main distinction of this approach is that it requires the execution of a set of structural transformations before the actual values can be introduced, which can, in certain cases, lead to different results. While a Boolean consistent FIS could be used for establishing the diagnostic criteria for any given disease, in this paper it is applied for determining the likelihood of peritonitis, as the leading complication of peritoneal dialysis (PD). Given that patients could be located far away from healthcare institutions (as peritoneal dialysis is a form of home dialysis) the proposed Boolean consistent FIS would enable patients to easily estimate the likelihood of them having peritonitis (where a high likelihood would suggest that prompt treatment is indicated), when medical experts are not close at hand.

## 1. Introduction

Fuzzy inference systems (FIS) allow decision makers to easily incorporate their own valuable experience into the decision-making process. More precisely, fuzzy logic is used to formally express expert knowledge in order to enable automated assessment and reasoning in a logically consistent manner akin to the way in which humans reason. Based on the premise that experience is better represented by linguistic means, fuzzy logic is an extremely appropriate tool for expressing domain knowledge without a need for a strong mathematical background. Consequently, fuzzy systems are nowadays being used more and more for modeling systems in a broad range of domains (including health care) and have repeatedly proven their efficiency.

However, no conventional fuzzy set theory (fuzzy logic, theory of fuzzy relations) is in the Boolean frame [1]. It is, therefore, proposed that Boolean consistent fuzzy logic, introduced in [2], should be used instead. The main distinction of the Boolean consistent approach (which is based on the Interpolative realization of Boolean algebra) is that it requires the execution of a set of structural transformations before the actual values can be introduced. This key difference between the conventional and Boolean consistent approaches can, in certain cases, lead to different results and ultimately to different decisions being made, as will be elaborated in Section 3.3.

While conventional FIS are regularly used in the field of medicine, this is the first time that a Boolean consistent FIS will be used in this domain. The main advantage of the proposed Boolean consistent FIS is that it preserves the transparency and interpretability inherent to fuzzy inference systems, while at the same time, introducing consistency in to the approach. While the proposed solution could be used for establishing the diagnostic criteria for any given disease, in this paper, for illustrative purposes, it will be applied for diagnosing peritonitis, which does in no way imply that it is only applicable to this problem.

Furthermore, this is the first time that either a conventional FIS or a Boolean consistent FIS is proposed for diagnosing peritonitis, as the leading complication of peritoneal dialysis (PD).

Peritoneal dialysis, as a form of home dialysis, is a specific form of treatment which requires the prior education of the patient to be able to self-administer this method. Patients are also educated in the clinical recognition of peritonitis (i.e., the inflammation of the peritonitis), as the most serious complication of peritoneal dialysis. If not recognized in time, or if inadequately treated, peritonitis can lead to serious complications and even death. Furthermore, severe and prolonged peritonitis can lead to peritoneal membrane failure; thus peritonitis is one of the main reasons for patients discontinuing PD and switching to hemodialysis. Consequently, it is very important to initiate treatment of PD-associated peritonitis as soon as possible.

However, given that a significant number of gastrointestinal diseases (including infectious and surgically related diseases) have similar clinical manifestations, wherein administration of antibiotic and analgesic therapy (particularly in the case of acute surgical diseases) may mask the clinical picture, it is necessary to have a clear differential diagnosis before starting therapy.

Since proper diagnostics may not always be readily available, it would be beneficial to establish a diagnostic approach that would enable patients to easily estimate the peritonitis likelihood in order to promptly initiate the necessary therapy. Therefore, an additional contribution of this paper is the introduction of a FIS incorporating medical experience, in the form of rules established by domain experts, which would be of assistance to patients when medical experts are not close at hand. Furthermore, because the rules are given in a natural (i.e., linguistic) form they are easier to express, validate, and modify by medical experts.

The conventional and Boolean consistent approaches will be elaborated and compared in order to clarify why the application of Boolean consistent fuzzy logic is preferred.

The paper is structured as follows: Section 2 provides an overview of the peritonitis likelihood estimation problem. The proposed approach is outlined in Section 3.  Section 4 is devoted to the experimental results and their analysis. Related work is given in Section 5 and finally, Section 6 concludes the paper and discusses future work.

## 2. Problem Description

Peritoneal dialysis is a renal replacement therapy method complementary with hemodialysis and renal transplantation. According to [3], peritonitis (i.e., inflammation of the peritoneum) remains a leading complication of peritoneal dialysis (PD) as around 18% of the infection-related mortality in PD patients is the result of peritonitis and even though less than 4% of peritonitis episodes result in death, peritonitis is a “contributing factor” to death in 16% of deaths on PD. Moreover, a number of potentially serious consequences of peritonitis (such as relapse, catheter removal, permanent transfer to hemodialysis, and death) are likely to occur if treatment is not initiated promptly. Consequently, peritonitis treatment (aimed at rapidly reducing the inflammation) should be initiated without delay.

Recommendations for the treatment of peritonitis, under the auspices of the International Society for Peritoneal Dialysis (ISPD), were first published in 1983 and are revised every five years. In accordance with these recommendations [3] peritoneal dialysis patients presenting with abdominal pain and cloudy effluent should be presumed to have peritonitis (this is confirmed by obtaining an effluent cell count, differential, and culture). While cloudy effluent usually indicates infectious peritonitis (even in the absence of abdominal pain), it can also be attributed to other causes. Hence, the abdomen should be drained and the effluent carefully inspected and sent for cell count with differential, Gram stain, and culture. An effluent cell count with white blood cells (WBC) more than 100/*μ*L (after a dwell time of at least 2 hours), with at least 50% polymorphonuclear neutrophilic cells, indicates the presence of inflammation, with peritonitis being the most likely cause.

On the other hand, it is also possible for peritonitis to be present even if the effluent is clear, and thus peritonitis should always be suspected in PD patients with abdominal pain. Furthermore, in a certain percentage of peritonitis cases it can be concluded, with considerable certainty, that the patient has peritonitis if the patient has a positive culture with a clear clinical manifestation or cloudy effluent, even if the number of leukocytes is not within the specified range (depending on the time of sampling). On the other hand, a clear clinical manifestation accompanied by a cloudy effluent or an increase in the number of leukocytes in the effluent, but with no agent isolated, can indicate sterile peritonitis (the sterile culture may be the result of improper sampling, previously administered antibiotic therapy, or the infection can be caused by specific agents which have a slow growth rate on standard microbiological media or require special cultivation).

## 3. Proposed Approach

Fuzzy logic, introduced by Zadeh [4], is envisaged to extend binary values 0 and 1, representing strict presence or absence of an entity, to any other real value in between, indicating an entity's relative presence or absence. These values, thus, indicate the degree to which an entity fulfills the characteristics of a given set. In addition, fuzzy logic is especially useful for working with linguistic variables, that is, variables whose values are words or sentences in a natural language [5]. The values of linguistic variables can be mapped to the [0,1] interval by the defining of suitable membership functions. On the other hand, domain experts can adequately express their knowledge and experience (which can sometimes be only in the form of vague and complex verbal statements), in a more natural way, and fuzzy set theory enables the transformation of such descriptions or statements into mathematical expressions.

As stated in [6] Zadeh proposed medical sciences as one of the first fields of application for fuzzy sets, as early as 1969. Furthermore, the author quotes Zadeh's thoughts on this subject: “it may be convenient to characterize a fuzzy set representing a disease [⋯] by its relation to various symptoms which in themselves are fuzzy in nature.”

### 3.1. Membership Functions

As defined in [4] a fuzzy set *A* in a universal set *X* is characterized by a membership function *μ*_*A*_(*x*) which associates with each point in *X* a real number in the interval [0,1], with the value of *μ*_*A*_(*x*) at *x* representing the “grade of membership” of *x* in *A*:

(1) $$\begin{array}{c} \mu_{A}\left(\;x\;\right):X⟶\left[\;0,1\;\right]. \end{array}$$

The larger the value of *μ*_*A*_(*x*), the higher the degree to which entity *x* fulfills the characteristics of *A*.

The shape of a membership function can be selected either intuitively (based on previous knowledge) or on the basis of the characteristics of the input-output data. Domain experts play a key role in this process as they possess an in-depth understanding of the domain, that is, the semantics of the data.

Specifically, the following input variables were used in this paper:

 F: fever,
 L: number of leukocytes,
 AP: abdominal pain,
 CE: cloudiness of effluent,
 MC: number of microorganisms.

It should be noted that “*cloudiness of effluent*” represents a linguistic variable with the following values: clear and slightly, considerably, or extremely cloudy.

**Figure 1:**
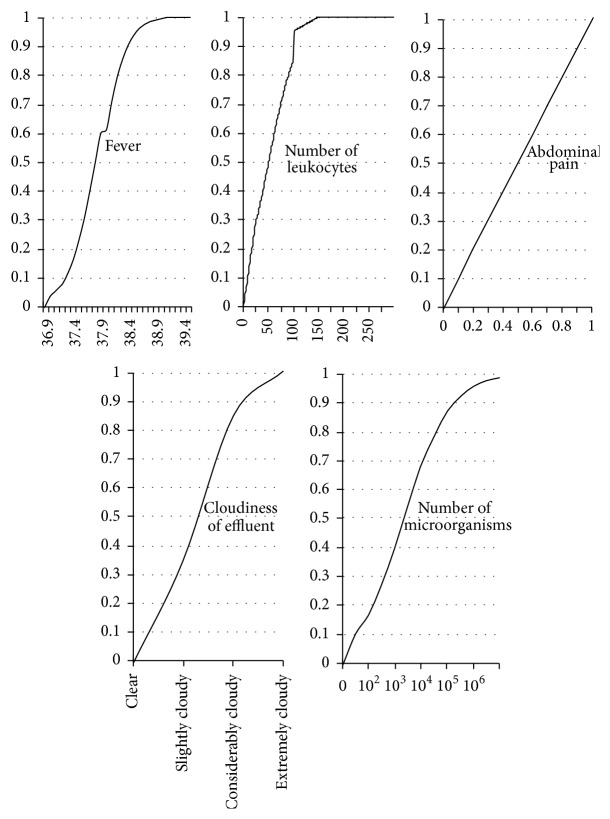
Membership functions of the input variables.

The corresponding membership functions (defined on the basis of a literature review and domain expertise) are depicted in Figure 1.

### 3.2. Fuzzy Inference Systems

One of the main applications of fuzzy logic today is found in fuzzy inference systems (FIS). According to [7], these systems are also known as fuzzy-rule-based systems, fuzzy models, fuzzy controllers, or fuzzy associative memories.

Essentially fuzzy inference systems are knowledge-based systems that use elements of fuzzy logic for modeling the relationship between the input space and the output space, that is, for inferring the outputs from the inputs.

According to [8], fuzzy logic is used to cast the verbal knowledge into a conventional mathematical representation. In other words, fuzzy logic is used to formally express expert knowledge in the form of fuzzy rules, that is, human comprehensible linguistic statements that enable the automation of the decision-making process.

Fuzzy rules are conditional statements of the form “*if X then Y*.” The* if* part of the rule (i.e., the premise) is a composition of fuzzy variables (characterized by appropriate membership functions) and fuzzy operators (corresponding to the common logical operators: “and,” “or,” and “not”). The* then* part (i.e., the consequence) is inferred from the “truth value,” or rule strength, of the premise (calculated on the basis of the values of the input variables).

Therefore, in addition to defining the membership functions, it is also necessary to choose the appropriate functions for the fuzzy conjunction (AND), fuzzy disjunction (OR), and fuzzy complement (NOT) operators. The choice of an operator depends on the problem to be solved, that is, the level of interaction between the elements that are being aggregated, as stated in [9].

In general the fuzzy inference process consists of three phases:

- Fuzzification in which the crisp values of the input variables are associated with membership degrees of the corresponding linguistic variables.
- Evaluation, that is, rule-based reasoning.
- Defuzzification in which the fuzzy result is transformed into a crisp output.

However, fuzzy inference systems differ mainly in the way in which the consequence is formulated, that is, whether the output variables are represented as fuzzy sets or not, as demonstrated by the two main FIS types: the well-known Mamdani [10] and Sugeno [11] models, respectively. In this paper the fuzzy sets are used only in the premise, and the output is defined as a crisp value indicating the likelihood that a patient has peritonitis:

PL: peritonitis likelihood.

Specifically, in accordance with the discussion in Section 2, in order to estimate the likelihood of peritonitis for PD patients the following rule was established by a domain expert:

 
*if*
   (MC* and* (AP* or* F* or* CE))
   
*or*
   (*not* MC* and* (AP* and* (CE* or* L)))
 
*then* PL

The rule can be interpreted as follows: a positive culture accompanied by a clinical manifestation or cloudy effluent OR a sterile culture but with abdominal pain and a cloudy effluent or an increase in the number of leukocytes in the effluent indicates a high probability of the patient having peritonitis. A high likelihood of peritonitis implies that it is necessary to promptly initiate treatment.

**Figure 2:**
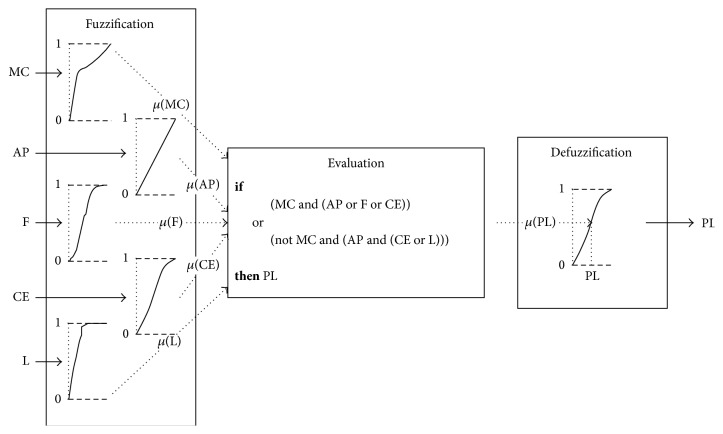
Fuzzy inference process for estimating peritonitis likelihood.

The specific fuzzy inference process, utilized in this paper, is depicted in Figure 2.

### 3.3. Boolean Consistent Fuzzy Logic

The main premise of this paper is that Boolean consistent fuzzy logic, introduced in [2], should be used in the evaluation of rules since no conventional fuzzy set theory (fuzzy logic, theory of fuzzy relation) is in the Boolean frame [1]. Furthermore, as stated in [12], the structures embedded in fuzzy set theories are usually less rich than the Boolean lattice of classical set theory.

To begin with, in order to justify why the new approach is proposed, it will be shown that conventional fuzzy set theory does not satisfy all Boolean axioms and theorems, foremost the axioms of excluded middle and contradiction.

The excluded middle axiom states that

(2) $$\begin{array}{c} \mu_{A}\left(\;x\;\right)\vee \neg \mu_{A}\left(\;x\;\right)=1, \end{array}$$

while the contradiction axiom states that

(3) $$\begin{array}{c} \mu_{A}\left(\;x\;\right)\wedge \neg \mu_{A}\left(\;x\;\right)=0. \end{array}$$

First, it should be noted that there is a variety of functions defined for the fuzzy operators. Ordinarily, a *t*-*norm* generalizes the AND operator, while the *s*-*norm* generalizes the OR operator (the *s*-norm must correspond to the chosen *t*-norm). Initially, the min and max functions were proposed for the AND or OR operators, respectively [4]. However, in this case, the result is only influenced by one of the elements (i.e., the dominant one) and thus they indicate no interaction [9]. In order to overcome this shortcoming and enable the membership values of both fuzzy sets to contribute to the result, other definitions of the operators were later proposed, with the product (for the AND operator) and the algebraic sum (for the OR operator) being the most commonly used. Finally, the fuzzy complement implies that if an entity has a property with a membership of *μ*_*A*_(*x*) then the absence of the property must have a membership of 1 − *μ*_*A*_(*x*). Thus, if an entity has a property with a membership of 0.2, then it could be said that the absence of the property has a membership of 0.8:

(4) μAx=0.2,1−μAx=0.8.

Consequently, the following examples ((5)–(8)) prove that the two previously stated axioms do not hold.

The* excluded middle* axiom is as follows:

(i) with the algebraic sum selected as the *s-*norm:
  (5) μAx∨¬μAxμAx+1−μAx−μAx∗1−μAx=0.2+0.8−0.16=0.84≠1,
(ii) or with the maximum selected as the *s*-norm:
  (6) μAx∨¬μAxmax⁡μAx,1−μAx=max⁡0.2,0.8=0.8≠1.

The same can be shown for the* contradiction* axiom:

(i) with the product selected as the *t*-norm:
  (7) μAx∧¬μAxμAx∗1−μAx=0.2∗0.8=0.16≠0,
(ii) or with the minimum selected as the *t*-norm:
  (8) μAx∧¬μAxmin⁡μAx,1−μAx=min⁡0.2,0.8=0.2≠0.

In light of the previous assertions the use of the interpolative realization of Boolean algebra (IBA) is proposed. As stated in [2] IBA is an illustrative name for the real-valued and/or [0, 1]-valued realization of Boolean algebra. It requires that a set of structural transformations be executed before the values can be introduced. In other words, only once the transformations have been conducted and the final structure established will the values be introduced and computed.

Any logical function can be uniquely transformed into a corresponding generalized Boolean polynomial (GBP) using IBA. It should be emphasized that the GBP maps a corresponding element of Boolean algebra into its value from the real unit interval [0, 1] at the* value* level, so that, contrary to other many valued and/or fuzzy approaches, a partial order on the value level is preserved [13].

The GBP is a polynomial whose variables are elements of the Boolean algebra and the operators are standard +, standard −, and generalized product ⊗. According to [14] the generalized product can be any function which maps [0,1]×[0,1]→[0,1] and is a subclass of the conventional fuzzy *t*-norm satisfying the nonnegativity axiom.

Furthermore, it is important to highlight that in this approach the following property always holds:

(9) $$\begin{array}{c} \mu_{A}\left(\;x\;\right)⊗\mu_{A}\left(\;x\;\right)=\mu_{A}\left(\;x\;\right). \end{array}$$

As a result, the excluded middle and contradiction axioms will hold ((10) and (11), resp.).

The* excluded middle* axiom is as follows:

(10) μAx∨¬μAxμAx+1−μAx−μAx⊗1−μAx=μAx+1−μAx−μAx⊗1+μAx⊗μAx=μAx+1−μAx−μAx+μAx=1.

The* contradiction* axiom is as follows:

(11) μAx∧¬μAxμAx⊗1−μAx=μAx⊗1−μAx⊗μAx=μAx−μAx=0.

It can, thus, be concluded that this main difference between the conventional and Boolean consistent approaches can, in certain cases, lead to different results (as will be shown in Section 4.3). Specifically, when the statement includes negation, the application of conventional fuzzy logic may lead to inadequate results due to the fact that it does not satisfy the excluded middle and contradiction axioms. On the other hand, in the Boolean consistent approach the atomic values are introduced only after the final structure has been established, thereby ensuring that all Boolean axioms are satisfied.

The main benefit of the proposed Boolean consistent FIS is that it preserves the transparency and interpretability inherent to fuzzy inference systems, while, at the same time, introducing consistency into the approach.

## 4. Experimental Results

In this section the proposed solution will be used for estimating peritonitis probability. Both the conventional and Boolean consistent FIS approaches will be presented and compared.

### 4.1. Data

The data was obtained from the clinical records of 156 patients who were in the PD program from 2001 to 2010 at the Military Medical Academy (located in Belgrade, Serbia). The Military Medical Academy is the largest military hospital in Serbia and South-Eastern Europe and one of the largest military hospitals in the world. It is a medical, educational, and scientific-research institution with an internationally acknowledged reputation in both civilian and military healthcare.

Out of the 156 available patient records, 123 patients had been diagnosed with peritonitis. The patients (74 males and 82 females) were between 18 and 85 years of age (mean age 59.7 ± 12.4 years).

As stated in their medical records, the diagnosis had been confirmed based on

1. clinical symptoms and signs of peritoneal inflammation (such as fever and abdominal pain),
2. cloudiness of effluent and elevation in number of leukocytes > 100 cells/*μ*L (over 50% PMN),
3. microbiological identification (Gram or culture). Dialysate specimens were obtained from all of the patients and examined for culture and resistance.

The remaining 33 patients had had some clinical indications (such as abdominal pain, fever, elevated number of leukocytes, and cloudy effluent) but the other diagnostic criteria had been absent. Thus, the symptoms could not be attributed to peritonitis and the diagnosis had not been confirmed. The presence (number and percentage of cases) of the relevant diagnostic criteria for the 123 and 33 patients, respectively, is given in Table 1.

Out of the 123 patients with peritonitis, 16 patients had sterile peritonitis; that is, they had culture-negative dialysates. The remaining patients tested positive for a number of different agents (Table 2).

The most severe episodes were caused by* Staphylococcus aureus*,* Enterococcus*, fungi (*Candida*), mixed organisms (*Gram-positive* and* Gram-negative*), and* Gram-negative* bacteria, while* Coagulase-negative staphylococcus* (CNS) leads to the mildest clinical forms.

### 4.2. Methods

The established rule (Section 3.2) could be mathematically expressed as

(12) $$\begin{array}{c} \left(\;MC\wedge \left(\;AP\vee \text{F}\vee CE\;\right)\;\right)\vee \left(\;\neg MC\wedge \left(\;AP\wedge \left(\;CE\vee \text{L}\;\right)\;\right)\;\right). \end{array}$$

As explained in Section 3.3, in order to transform a Boolean function into a generalized Boolean polynomial (GBP), the first step is to assess its structure. A detailed description of this transformation is given in [13]. In this particular case the following transformation steps have been taken ((13)-(14)).

In [15] a software, jFuzzyIBATranslator, is implemented which can transform any logical expression into the corresponding GBP. The software could be exploited to bypass the need for conducting these transformations manually, thereby rendering the proposed solution more approachable and convenient for nontechnical users.

After the transformation has been accomplished (preferably automatically) focus is transferred to the value level, by choosing the standard product as an adequate operator for the generalized product and then inputting the membership values:

(13) MC∧AP∨F∨CE∨¬MC∧AP∧CE∨L⊗=MC∧AP∨F∨CE⊗︸1+¬MC∧AP∧CE∨L⊗︸2−MC∧AP∨F∨CE⊗⊗¬MC∧AP∧CE∨L⊗︸3,1  MC∧AP∨F∨CE⊗=MC⊗AP∨F∨CE⊗=MC⊗AP+F+CE−AP⊗F−AP⊗CE−F⊗CE+AP⊗F⊗CE=MC⊗AP+MC⊗F+MC⊗CE−MC⊗AP⊗F−MC⊗AP⊗CE−MC⊗F⊗CE+MC⊗AP⊗F⊗CE,2  ¬MC∧AP∧CE∨L⊗=1−MC⊗AP∧CE∨L⊗=1−MC⊗AP⊗CE∨L⊗=1−MC⊗AP⊗CE+L−CE⊗L=1−MC⊗AP⊗CE+AP⊗L−AP⊗CE⊗L=AP⊗CE+AP⊗L−AP⊗CE⊗L−MC⊗AP⊗CE−MC⊗AP⊗L+MC⊗AP⊗CE⊗L,3  MC∧AP∨F∨CE⊗⊗¬MC∧AP∧CE∨L⊗=MC⊗AP+MC⊗F+MC⊗CE−MC⊗AP⊗F−MC⊗AP⊗CE−MC⊗F⊗CE+MC⊗AP⊗F⊗CE⊗AP⊗CE+AP⊗L−AP⊗CE⊗L−MC⊗AP⊗CE−MC⊗AP⊗L+MC⊗AP⊗CE⊗L=MC⊗AP⊗CE+MC⊗AP⊗L−MC⊗AP⊗CE⊗L−MC⊗AP⊗CE−MC⊗AP⊗L+MC⊗AP⊗CE⊗L+MC⊗F⊗AP⊗CE+MC⊗F⊗AP⊗L−MC⊗F⊗AP⊗CE⊗L−MC⊗F⊗AP⊗CE−MC⊗F⊗AP⊗L+MC⊗F⊗AP⊗CE⊗L+MC⊗CE⊗AP+MC⊗CE⊗AP⊗L−MC⊗CE⊗AP⊗L−MC⊗CE⊗AP−MC⊗CE⊗AP⊗L+MC⊗CE⊗AP⊗L−MC⊗AP⊗F⊗CE−MC⊗AP⊗F⊗L+MC⊗AP⊗F⊗CE⊗L+MC⊗AP⊗F⊗CE+MC⊗AP⊗F⊗L−MC⊗AP⊗F⊗CE⊗L−MC⊗AP⊗CE−MC⊗AP⊗CE⊗L+MC⊗AP⊗CE⊗L+MC⊗AP⊗CE+MC⊗AP⊗CE⊗L−MC⊗AP⊗CE⊗L−MC⊗F⊗CE⊗AP−MC⊗F⊗CE⊗AP⊗L+MC⊗F⊗CE⊗AP⊗L+MC⊗F⊗CE⊗AP+MC⊗F⊗CE⊗AP⊗L−MC⊗F⊗CE⊗AP⊗L+MC⊗AP⊗F⊗CE+MC⊗AP⊗F⊗CE⊗L−MC⊗AP⊗F⊗CE⊗L−MC⊗AP⊗F⊗CE−MC⊗AP⊗F⊗CE⊗L+MC⊗AP⊗F⊗CE⊗L=0.

Finally,

(14) MC∧AP∨F∨CE∨¬MC∧AP∧CE∨L⊗=MC⊗AP+MC⊗F+MC⊗CE−MC⊗AP⊗F−MC⊗AP⊗CE−MC⊗F⊗CE+MC⊗AP⊗F⊗CE+AP⊗CE+AP⊗L−AP⊗CE⊗L−MC⊗AP⊗CE−MC⊗AP⊗L+MC⊗AP⊗CE⊗L−0=MC⊗AP+MC⊗F+MC⊗CE+AP⊗CE+AP⊗L−MC⊗AP⊗F−2∗MC⊗AP⊗CE−MC⊗F⊗CE−AP⊗CE⊗L−MC⊗AP⊗L+MC⊗AP⊗F⊗CE+MC⊗AP⊗CE⊗L.

This rule can also be evaluated by applying the rules of* conventional* fuzzy logic:

(15) MC∧AP∨F∨CE∨¬MC∧AP∧CE∨L=MC∧AP∨F∨CE+¬MC∧AP∧CE∨L−MC∧AP∨F∨CE∗¬MC∧AP∧CE∨L=MC∗AP+MC∗F+MC∗CE−MC∗AP∗F−MC∗AP∗CE−MC∗F∗CE+MC∗AP∗F∗CE+AP∗CE+AP∗L−AP∗CE∗L−MC∗AP∗CE−MC∗AP∗L+MC∗AP∗CE∗L−MC∗AP+MC∗F+MC∗CE−MC∗AP∗F−MC∗AP∗CE−MC∗F∗CE+MC∗AP∗F∗CE∗AP∗CE+AP∗L−AP∗CE∗L−MC∗AP∗CE−MC∗AP∗L+MC∗AP∗CE∗L.

### 4.3. Result Analysis

The validity of the proposed approaches was assessed by comparing the actual diagnoses from the 156 available patient records with the results (i.e., peritonitis likelihood: PL) obtained by inputting the relevant data (i.e., membership values) into (14) and (15), for the Boolean consistent FIS and the conventional FIS, respectively. The test data was divided into two sets: 123 patients with confirmed peritonitis and 33 patients who had clinical indications, but whose symptoms were found to be caused by other diseases.

Out of the 123 patients diagnosed with peritonitis, both of the proposed approaches estimated the probability of peritonitis to be more than 60% in 114 cases (92.68% of this sample) with the probability being more than 75% in 106 of these cases (86.18% of the sample).

For the remaining 33 patients, both approaches estimated the probability to be less than 37.5%. Moreover, the estimated probability could be considered insignificant (less than 4%) for 25 patients (75.76% of this sample).

Both approaches estimated the same likelihood in 29/123, that is, 33/33 cases. However, the Boolean consistent FIS dominated the conventional FIS (i.e., it indicated a higher peritonitis likelihood) in the remaining 94/123 cases (76.42% of the sample) and with more than a 10% difference in likelihood in 36 cases (29.27% of the sample). The results have thus shown that these two approaches might not always lead to the same conclusions. Moreover, the Boolean consistent FIS constantly outperformed the conventional FIS as was expected in light of the previous discussion (Section 3.3).

In addition, the most significant discrepancy between these two approaches is depicted in Table 3. Based on the severity of their symptoms (columns 1–5) it could be asserted, with considerable certainty, that these 12 patients have peritonitis and that medical treatment should be initiated immediately. The 100% value in column 6 of Table 3 shows that the Boolean consistent FIS correctly assessed the severity (the diagnosis had also been confirmed in their medical records). However, the results obtained by applying the conventional FIS (column 7 of Table 3) underestimate the likelihood by 21.25% on average.

For clarification purposes, the values in Table 3 (columns 6 and 7) for the last row were calculated, in accordance with (14) and (15), respectively, with the following membership values:

(i) F = *μ*_F_(*x*) = 0.99817,
(ii) L = *μ*_L_(*x*) = 1,
(iii) AP = *μ*_AP_(*x*) = 1,
(iv) CE = *μ*_CE_(*x*) = 1,
(v) MC = *μ*_MC_(*x*) = 0.67863.

## 5. Related Work

In the past couple of decades FIS have proven to be an extremely efficient decision support system in a wide range of different domains (from industrial engineering through finance and health care).

Most of the papers dealing with peritonitis diagnosis using computational and mathematical methods are focused on the application of statistical methods [16–19].

The application of a Boolean consistent FIS is proposed for the first time in the medical domain. In addition the conventional FIS has not, thus far, been applied for diagnosing peritonitis.

The justification for the introduction of the proposed approach can be found in the numerous works proposing the use of fuzzy logic and FIS for diagnosing various diseases (see [5, 20–25] for a detailed survey).

Among the many approaches proposing FIS in medicine the following diseases have been studied the most:

 
*Heart Disease*
  - A fuzzy expert system which determines the risk of a patient developing a coronary heart disease in the next ten years and recommends three outputs (normal living, diet, or drug treatment) is proposed in [26].
  - An automatically generated fuzzy rule-based decision support system for diagnosis of coronary artery disease is presented in [27]. The system is automatically generated from an initial annotated dataset.
  - The hypertension risk for different age and gender groups is determined in [28].
 
*Cancer*
  - The percentage of possibility of prostate cancer risk is predicted in [29] in order to determine whether a biopsy should be performed.
  - Fuzzy rules are used in [30] to obtain a breast cancer risk prognosis.
  - The FIS proposed in [31] accepts symptoms as its input and provides the confirmation of lung cancer, and its stage, as the output.
 
*Respiratory Disorders*
  - The work presented in [32] is aimed at detecting asthma in its early stages. The authors propose two FIS modules for determining the degree of possibility of asthma. The first uses only the relevant symptoms as inputs and the second represents a linear combination of the symptom-based FIS and a FIS that exploits laboratory results pertaining to lung function.
  - An automated system for diagnosing the severity of asthma is introduced in [33].

In addition FIS has also been used for the diagnosis of malaria [34, 35], migraine [36], back pain [37], impotence [38], and liver disorders [39] and for detecting possible complications during anesthesia [40, 41].

## 6. Conclusions

Fuzzy logic has been widely used to assist decision makers in a number of different domains. It is used to formally express expert knowledge (which can usually only be expressed as vague and complex verbal statements) in the form of fuzzy rules, thereby enabling the automation of the decision-making process.

Fuzzy inference systems have, to date, proven to be extremely useful and efficient in the field of medicine and health care. This is the first time that FIS is proposed for diagnosing peritonitis, as the leading complication of peritoneal dialysis (PD).

If peritonitis is not recognized in time, or if it is inadequately treated, it can lead to serious complications and even death. Since patients could be located far away from healthcare institutions (as peritoneal dialysis is a form of home dialysis) it would be beneficial to establish a diagnostic approach that would enable patients to easily estimate the likelihood of them having peritonitis in order to promptly initiate the necessary treatment. Given that medical expertise is incorporated into the system, in the form of rules established by domain experts, the proposed FIS could assist patients when medical experts are not close at hand. A high likelihood of peritonitis would, then, suggest that prompt treatment is indicated.

However, since no conventional fuzzy set theory is in the Boolean frame it is also proposed that Boolean consistent fuzzy logic could be used in the evaluation of rules. The main distinction of this approach is that it requires the execution of a set of structural transformations before the actual values can be introduced. It has been demonstrated and clarified why this key difference between the conventional and Boolean consistent approaches can, in certain cases, lead to different results.

By using a Boolean consistent FIS not only would negation be treated more adequately but, at the same time, all Boolean logic axioms would hold.

This is the first time that Boolean consistent FIS is proposed in the medical domain. While the proposed solution could be used for establishing the diagnostic criteria for any given disease, in this paper, for illustrative purposes, it was applied for determining the likelihood of peritonitis.

The main advantage of an improved Boolean consistent FIS is that it preserves the transparency and interpretability inherent to fuzzy inference systems, while, at the same time, introducing consistency into the approach.

The experimental results show that the Boolean consistent FIS constantly outperformed the conventional FIS; that is, it gave the same or even a better estimation for all test cases (and with more than a 10% difference in peritonitis likelihood in 23.08% of the entire sample).

Further work would be aimed at exploring the effects of introducing more than one fuzzy set for describing the variables.

## Figures and Tables

**Table 1:** Clinical presentation of PD peritonitis.

Criterion		Confirmed		Not confirmed	
		#	Percent	#	Percent
Fever	(F)	48	(39.02%)	16	(48.48%)
Number of leukocytes	(L)	123	(100%)	11	(33.33%)
Abdominal pain	(AP)	108	(87.80%)	26	(78.79%)
Cloudiness of effluent	(CE)	114	(92.68%)	14	(42.42%)
Microbiological culture	(MC)	107	(86.99%)	0	(0%)

**Table 2:** Distribution of microbial agents.

Agent	#	Percent
Gram-positive		
*Coagulase-negative staphylococcus*	50	(46.73%)
*Staphylococcus aureus*	9	(8.41%)
*Streptococcus viridans*	9	(8.41%)
*Enterococcus*	6	(5.61%)
*Corynebacterium*	5	(4.67%)
	**79**	**(73.83%)**
Gram-negative		
*Escherichia Coli*	7	(6.54%)
*Acinetobacter*	6	(5.61%)
*Klebsiella*	3	(2.80%)
*Stenotrophomonas*	2	(1.87%)
*Pseudomonas*	1	(0.93%)
*Enterobacter*	1	(0.93%)
*Proteus mirabilis*	1	(0.93%)
*Morganella*	1	(0.93%)
	**22**	**(20.56%)**
Mixed	4	(3.74%)
Fungi (*Candida*)	2	(1.87%)

**Table 3:** Result comparison.

F	L	AP	CE^*∗*^	MC	PL Consistent FIS	PL Conventional FIS
38.2	168	1	EC	6.1 · 10^4^	**100.00%**	**78.19%**
38.9	186	1	EC	7 · 10^4^	**100.00%**	**78.19%**
38.2	198	1	EC	1.4 · 10^5^	**100.00%**	**83.39%**
37.7	164	1	EC	8.3 · 10^4^	**100.00%**	**78.19%**
39.2	258	1	EC	2 · 10^4^	**100.00%**	**75.19%**
39.1	187	1	EC	8 · 10^5^	**100.00%**	**88.66%**
38.4	176	1	EC	3.3 · 10^5^	**100.00%**	**83.39%**
38.1	163	1	EC	5.1 · 10^5^	**100.00%**	**88.66%**
38.4	164	1	EC	3 · 10^5^	**100.00%**	**83.39%**
38.1	157	1	EC	10^6^	**100.00%**	**92.82%**
38.7	205	1	EC	6.5 · 10^5^	**100.00%**	**88.66%**
39.3	298	1	EC	5.9 · 10^4^	**100.00%**	**78.19%**

^*∗*^The EC value in the table stands for Extremely Cloudy.
